# P-987. Outcomes of a Program to Integrate the National HIV Curriculum into Health Profession Programs

**DOI:** 10.1093/ofid/ofae631.1177

**Published:** 2025-01-29

**Authors:** Trisha Melhado, Nathaniel Teplitskly, Suyen Schneegans, Stacey Griner, Waridibo Allison

**Affiliations:** UNTHSC, Fort Worth, Texas; UNTHSC, Fort Worth, Texas; UNTHSC, Fort Worth, Texas; UNTHSC, Fort Worth, Texas; UNTHSC, Fort Worth, Texas

## Abstract

**Background:**

The HIV epidemic is ongoing, but the HIV-specific workforce is decreasing. Targeted Access Knowledge and Education on HIV for Health Professions Programs (TAKE on HIV for Health Professions Programs) focuses on the integration of the National HIV Curriculum (NHC) into health professions programs (HPPs) to encourage health professional students/trainees (learners) to enter the HIV workforce and incorporate HIV prevention and care into their practice. This study details integration outcomes.

**Methods:**

The NHC consists of six modules: Screening and Diagnosis, Basic HIV Primary Care, Antiretroviral Therapy, Co-Occurring Conditions, Prevention of HIV, and Key Populations. The number and types of HPPs integrating the NHC were assessed along with the number of learners using the NHC. Students were stratified into Learning Groups (LGs) based on their HPP discipline to track NHC module use and capture knowledge scores. Post integration cycle, a survey assessed learners’ intent to apply the knowledge gained from the NHC into their career (Likert scale: 1-not at all to 5-a great deal). Faculty indicated their intent to continue NHC integration from very unlikely to very likely (5-point Likert scale). Data were analyzed using descriptive statistics.

**Results:**

From September 2023 to April 2024, 15 unique student LGs with 591 learners were integrating the NHC overseen by 14 faculty members at 12 HPPs. Learner NHC enrollment by discipline was as follows: 353 Pharmacy students, 72 Physician Assistant students, 50 Nursing students, 103 Medical Residents, and 13 Occupational Therapy students. The Co-Occurring Conditions NHC module was used the most (1,689 lessons completed) whereas the Key Populations module was used the least (342 lessons completed). Overall mean NHC module scores ranged from 88% to 95% with the Antiretroviral Therapy module and Key Populations module scoring highest and lowest, respectively. Mean student score for intent to apply NHC knowledge = 3.80 (N=175). All integrating faculty indicated they were "very likely" to implement the NHC in future courses.

**Conclusion:**

TAKE on HIV program outcomes indicate successful facilitation of NHC integration into HPPs. The NHC is an effective online mechanism to educate health professional learners and to bolster the HIV workforce pipeline.

**Disclosures:**

**All Authors**: No reported disclosures

